# Experimental Investigation of Temperature Polarization near Membrane Surface During Air Gap Membrane Distillation Processes

**DOI:** 10.3390/membranes15060185

**Published:** 2025-06-18

**Authors:** Lianqi Jing, Jiaqi Sun, Yaoling Zhang, Jiaming Chen, Fei Guo

**Affiliations:** School of Energy and Power Engineering, Dalian University of Technology, Dalian 116024, China; 3066027602@mail.dlut.edu.cn (L.J.); sunjiaqizg@mail.dlut.edu.cn (J.S.); 71910003@mail.dlut.edu.cn (Y.Z.); cjm00000@mail.dlut.edu.cn (J.C.)

**Keywords:** membrane distillation, temperature polarization, transmembrane flux, thermocouple, temperature polarization factor, near membrane surface, effective ratio for mass transfer coefficient

## Abstract

Temperature polarization is a critical factor influencing the performance of membrane distillation. The presence of temperature polarization causes the temperature of the fluid near the membrane surface to be different from that in the bulk region, reducing the effective temperature difference across the membrane and thus diminishing the transmembrane mass transfer driving force. This study investigates the monitoring of temperature polarization and its effects on the transmembrane mass transfer performance in a typical air gap membrane distillation system. A set of thermocouples within a feed module were employed to monitor and capture the development of the temperature polarization profile. The test results reveal that temperature polarization reduces the effective temperature difference across the membrane, leading to a certain difference between the theoretical estimation and experimental values of the mass transfer coefficient across the porous membrane. To address this issue, the temperature polarization factor was further analyzed as a metric to quantify the impact of temperature polarization on the transmembrane flux in membrane distillation, with a detailed discussion of its range and implications.

## 1. Introduction

In response to the scarcity of freshwater resources, various methods for freshwater production have been proposed, such as the use of photothermal evaporators [1], atmospheric water harvesting (AWH) [2], and membrane desalination (MD). These developments have also led to the emergence of a variety of energy storage devices [3,4]. Membrane distillation is a membrane separation technology that integrates membrane-based processes with thermal evaporation at interfaces. In the MD process, a hydrophobic porous membrane acts as a barrier separating the feed (to be separated) and the condensing fluid. The feed and the condensing fluid circulate on opposite sides of the membrane. In membrane distillation, a temperature gradient across the membrane creates a vapor pressure difference that drives water evaporation at the hot side. The vapor migrates through the membrane pores and condenses on the cold side.

Transmembrane flux is a key parameter for evaluating the mass transfer performance of MD systems. Many studies have aimed at optimizing conditions to achieve higher transmembrane flux. Based on the diffusion mechanism of water molecules through pores, the type of transport can be categorized into molecular diffusion, Knudsen diffusion, or transition diffusion [5]. Theoretical flux values can be calculated using these diffusion mechanisms [6]. However, previous studies have shown that theoretical flux values often differ significantly from experimental results due to various factors, including the physical properties of the hydrophobic porous membrane (porosity, tortuosity, thickness) and operational parameters.

In MD, temperature polarization describes the temperature difference between the membrane surface and the bulk. It is a critical factor contributing to the reduction in transmembrane flux [7]. The hot and cold sides of the hydrophobic porous membrane are in direct contact with the feed and the condensing fluid (usually water), respectively. Water in the feed evaporates at the hot side of the membrane and condenses on the cold side, leading to the development of thermal boundary layers on both sides of the membrane. As a result, the temperature at the membrane is different from that of the bulk liquid, reducing the effective temperature difference across the membrane compared to the bulk temperature difference. According to the Antoine equation, the vapor pressure of water is exponentially related to temperature [8,9]. A reduction in the temperature of the liquid near the membrane surface lowers the vapor pressure, causing the loss of the mass transfer driving force. The magnitude of the temperature gradient across the membrane determines the intensity of temperature polarization, which affects both the transmembrane flux and thermal efficiency in MD systems [10,11]. Studies have shown that the non-isothermal boundary layer near the membrane surface can reduce the driving force of mass transfer, resulting in a significant decrease in transmembrane flux [12].

Temperature polarization monitoring methods can be categorized into contact-based and noncontact-based approaches. Hijaz et al. used micro-thermocouples in a direct contact membrane distillation (DCMD) module to measure the temperatures on the hot and cold sides at the membrane surface and above the membrane [13]. Their analysis demonstrated that feed temperature and flow rate significantly influence transmembrane flux, with polarization being more pronounced at low flow rates and high feed temperatures. Their study also found that polarization is the most severe near the membrane surface, and its intensity is greater at the hot feed inlet than at the outlet. Ali et al. employed 16 fixed thermal resistors in a DCMD module to measure temperatures in the bulk fluid and membrane surface for both the hot feed and cooling water [14]. Their results revealed that the temperature polarization coefficient increases with feed flow rate and temperature. Transmembrane flux increases nonlinearly with the polarization coefficient and grows rapidly when the coefficient exceeds 0.84. Additionally, increasing feed concentration amplifies the temperature gradient, making polarization more significant.

Santoro et al. embedded temperature-sensitive molecular sensors in PVDF membranes for temperature monitoring on the membrane surface in DCMD, using infrared cameras for real-time temperature analysis [15]. Their findings showed a temperature gradient with membrane surface temperature decreasing along the feed flow direction. This gradient resulted in significantly lower membrane surface temperatures near the outlet compared to the inlet feed temperature. Tamburini et al. used thermochromic liquid crystals and digital image analysis to characterize temperature polarization [16]. Dalle et al. established an optical monitoring method to measure the temperature gradient near the membrane surface in AGMD [17].

Xu et al. investigated the effects of membrane properties on AGMD [18]. Their results showed that the transmembrane flux can be increased by using membranes with low thermal conductivity and high porosity. During this process, water vaporizes on the hot-side surface of the membrane, absorbing heat, and then condenses on the cold-side surface, releasing heat. Temperature polarization occurs during this heat and mass transfer, leading to a deviation in the membrane surface temperatures from the corresponding bulk temperatures. This reduction in driving force results in a decrease in transmembrane flux.

Increasing the porosity enhanced the diffusion rate of water vapor, allowing more heat to be removed from the hot side, thereby intensifying temperature polarization. However, the resulting increase in water yield outweighs the negative impact of enhanced temperature polarization. In addition, using membrane materials with low thermal conductivity reduces heat loss between the hot and cold sides, thereby mitigating temperature polarization [19].

Temperature polarization is a typical near-interface phenomenon, and altering the flow state of the liquid near the membrane surface is an effective way to mitigate it. Increasing the feed flow rate or using turbulence promoters, such as support meshes or corrugated feed channels, can enhance flow velocity and induce turbulence [20,21], thereby improving convective heat transfer near the membrane surface and suppressing temperature polarization. Adding independent cylindrical or rectangular baffles in the feed channel creates disturbances as the liquid flows [22,23]. Reducing the channel height in DCMD systems brings the bulk fluid closer to the membrane surface, significantly enhancing near-interface heat transfer and improving system performance [24]. Experimental studies have also shown that corrugated feed channels outperform commercial support meshes under similar operating conditions, providing superior flux and water recovery rates [25].

Multiphase flow is another effective method for mitigating polarization effects. Introducing bubbles into the feed generates gas–liquid two-phase flow, which reduces polarization effects [26,27,28]. Previous studies by our group demonstrated that adding solid particles to the feed or modifying the membrane surface can effectively suppress temperature polarization [29,30,31].

This study focuses on the monitoring of temperature polarization in MD and its impact on transmembrane mass transfer performance. A lab-scale air gap membrane distillation (AGMD) setup was constructed, where contact-based thermocouples were used to measure the temperatures near the membrane surface and in the bulk liquid. The effects of feed properties on temperature polarization were investigated experimentally, along with the influence of temperature polarization on MD performance. A new approach was used involving the use of hot-side (***α***) and cold-side (***β***) temperature polarization factors to quantify the impact of temperature polarization on mass transfer performance, with a detailed analysis of their range in common MD operating conditions. To account for the combined effects of temperature polarization on both the hot and cold sides, the effective ratio for the mass transfer coefficient (***η***) is proposed to represent the intensity to which the overall mass transfer performance of the system is affected. Furthermore, the correlation between ***η*** and temperature polarization factors is analyzed in this study.

## 2. Experimental Section

### 2.1. AGMD Module

The hydrophobic porous membrane used in this work was made of polytetrafluoroethylene (PTFE) with a pore size of 0.22 μm (FPB022N, Membrane Solutions, Shanghai, China). The surface morphology of the porous membrane was characterized using a tungsten filament scanning electron microscope (SEM, QUANTA 450, FEI, Hillsboro, OR, USA). The water contact angle and porosity of the membrane were measured using a water contact angle goniometer (YIKE-360A, Chengde Yike Testing Instrument Co., Ltd., Chengde, China) and the gravimetric method, respectively. The membrane porosity was 75 ± 5%, and the water contact angle was 145 ± 5°. The membrane thickness was measured as 40 ± 5 μm using a digital micrometer (211-101F, Guilin Guanglu Measuring Instrument Co., Ltd., Guilin, China). Micro-thermocouples (TT-T-30, Xinghua Suma Electric Instrument Co., Ltd., Xinghua, China) and a temperature scanner (SG-MD814-81-23-2H2L-P, Shanghai Shangge Sensor Technology Co., Ltd., Shanghai, China) were used to measure the temperature. A magnetic pump (MP-15R, Dongguan Guangquan Pump Industry Co., Ltd., Dongguan, China) was employed to ensure the smooth circulation of the solution. The flow rate was controlled by a flow meter (LZ M-4T, Yuyao Jintai Instrument Co., Ltd., Yuyao, China). An electronic balance (BT3000, Shanghai Yusheng Weighing Apparatus Co., Ltd., Shanghai, China) was used to measure water production.

An AGMD module was employed for the experiments. The feed and coolant channels were machined into an acrylic plate with dimensions of 475 mm × 150 mm × 20 mm. The membrane was fixed between two acrylic mesh plates to ensure flatness during the experiments. The air gap thickness was set at 2 mm, and the condensation plate was made of stainless steel with a thickness of 1 mm. All components within the module were sealed using 2 mm silicone gaskets. To ensure uniform pressure distribution during the tightening process, two stainless steel plates (thickness: 1 mm) were used as outer frames for the module.

### 2.2. MD Experimental System

**Figure 1:**
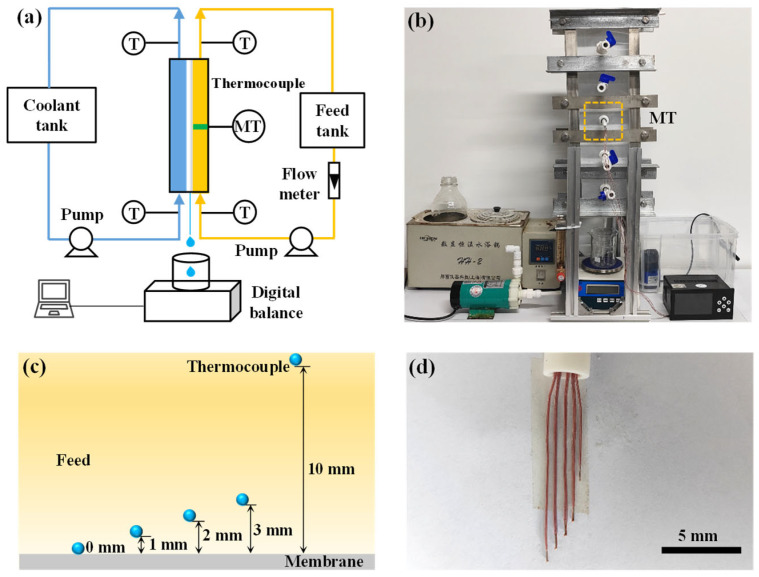
Experimental setup and measurement schematic for investigating temperature polarization in MD. (**a**) Schematic of experimental system: setup includes feed tank, coolant tank, thermocouples for temperature monitoring (T), digital mass balance for permeate measurement, and flow meter for feed flow control. Multiple thermocouples (MTs) are equipped with embedded thermocouples for localized temperature measurements near membrane surface. The blue arrows and yellow arrows indicate the flow directions of coolant flow and feed flow, respectively. (**b**) Photograph of experimental apparatus. (**c**) Schematic diagram illustrating spatial distribution of thermocouples near feed-side membrane surface, positioned at distances of 0 mm, 1 mm, 2 mm, and 3 mm to assess temperature polarization effect. (**d**) Closeup image of thermocouple array embedded near membrane surface, demonstrating precise positioning to capture temperature gradients.

The AGMD module was vertically oriented during the experiments (Figure 1a,b). To ensure the feed filled the module effectively, both the feed and coolant were circulated in a bottom-to-top flow configuration. The feed was a 35 g·L^−1^ NaCl solution, and the coolant was tap water (TDS = 100 ± 20 mg·L^−1^). The temperature of the tap water was maintained at 299 ± 2 K. The transmembrane flux decreases with increasing feed salinity due to reduced water activity at high salt concentrations. This investigation focuses on temperature and concentration polarizations during MD. A similar trend is observed under different initial salt concentrations. Both temperature and concentration polarizations occur simultaneously, and temperature polarization is the dominant factor affecting transmembrane flux [32]. An aqueous NaCl solution with a concentration of 35 g·L⁻^1^, similar to typical seawater salinity, was used to simulate seawater treatment. After being heated in a water bath, the feed was driven by a magnetic pump, and its flow rate was controlled using a rotameter. In the experiment, the product’s water quality was measured using an electronic balance, with weight data recorded every minute by a computer. The transmembrane flux was calculated using the following equation [29]:

(1) $$J=\frac{∆m}{A_{m}∆t}$$

where **Δ*m*** is the mass of the permeate (kg), ***A*_m_** is the effective membrane area (m^2^), and **Δ*t*** is the time interval (h).

### 2.3. Arrangement of Thermocouples

In this study, contact-type thermocouples were used to measure the temperature. The typical operating temperature range of feeds in membrane distillation is 313 K to 363 K. T-type thermocouples, with high sensitivity (temperature range: −73 K to 533 K; accuracy: ±0.1 K), were employed to measure the temperature gradient within the module. To minimize the influence of the thermocouple diameter on the flow of the near-membrane interface feed, micro-thermocouples with a wire diameter of approximately 1 mm were selected.

As shown in Figure 1c, five thermocouples were mounted on a single holder to measure the temperature of the feed at different heights above the membrane. The bulk feed temperature was defined as the temperature measured at 10 mm above the membrane, corresponding to half the height of the feed channel.

Due to the small diameter of the thermocouples, slight bending could occur as the feed flowed within the module. To ensure consistent placement, the five thermocouples were fixed together during the setup phase (as shown in Figure 1d). Before the experiments, the thermocouples were calibrated at fixed temperature points to minimize measurement errors.

To avoid disturbances caused by high turbulence at the inlet and outlet of the feed, the measurement points were located at the middle section of the module. A temperature recorder was used to collect data, with measurements taken every second over a 40 min duration for each test. The average value of the recorded data was used as the final result.

## 3. Theoretical Section

The mass transfer coefficient (***B***) is a key parameter characterizing the mass transfer performance of an MD system. It can be determined using the transmembrane flux and the vapor pressure difference across the membrane [29]:

(2) $$J_{w}=B{(P}_{f}-P_{c})$$

where ***P*_f_** (Pa) and ***P*_c_** (Pa) are the vapor pressures at the feed and coolant temperatures, respectively.

For feed, the vapor pressure can be calculated using the Antoine equation [29]:

(3) $$P=exp(23.1964-\frac{3816.44}{T-46.13})$$

where ***T*** (K) is the water temperature, and ***P*** (Pa) is the saturated vapor pressure under this temperature.

To describe and evaluate the intensity of temperature polarization, we introduce correction coefficients into the equation, including the hot-side temperature polarization factor (***α***), the cold-side temperature polarization factor (***β***), and the effective ratio (***η***) for the mass transfer coefficient. In previous studies, the bulk temperature was commonly used to calculate transmembrane flux and the mass transfer coefficient. By combining Equations (2) and (3), the following expression can be derived:

(4) $$J_{w}=B_{b}\left[\exp\left(23.1964-\frac{3816.44}{T_{f,b}-46.13}\right)-\exp\left(23.1964-\frac{3816.44}{T_{c,b}-46.13}\right)\right]$$

where ***T*_f,b_** represents the bulk temperature of the feed (hot side), and ***T*_c,b_** represents the bulk temperature of the coolant (cold side). ***B*****_b_** represents the mass transfer coefficient calculated using the bulk temperatures of the feed and coolant, ***T*_f,b_** and ***T*_c,b_**. However, due to the presence of temperature polarization, the membrane surface temperature differs from the bulk temperature. Therefore, using the membrane surface temperature yields more accurate results. The corresponding expression is given below:

(5) $$J_{w}=B_{s}\left[\exp\left(23.1964-\frac{3816.44}{T_{f,s}-46.13}\right)-\exp\left(23.1964-\frac{3816.44}{T_{c,s}-46.13}\right)\right]$$

where ***T*_f,s_** represents the temperature of the feed (hot side) at the membrane surface, and ***T*****_c,s_** represents the temperature of the coolant (cold side) at the membrane surface. ***B*****_s_** represents the mass transfer coefficient calculated using the membrane surface temperatures of the feed and coolant, ***T*_f,s_** and ***T*_c,s_**.

The hot-side temperature polarization factor (***α***) is defined as ***α*** = ***T*_f,s_**/***T*_f,b_**, and the cold-side temperature polarization factor (***β***) is defined as ***β*** = ***T*_c,s_**/***T*_c,b_**. Substituting these into Equation (5) yields the following:

(6) $$J_{w}=B_{s}\left[\exp\left(23.1964-\frac{3816.44}{{\alpha T}_{f,b}-46.13}\right)-\exp\left(23.1964-\frac{3816.44}{{\beta T}_{c,b}-46.13}\right)\right]$$

The value of ***α*** ranges between 0 and 1, while ***β*** is greater than 1. When temperature polarization decreases, the ***α*** value increases, and the ***β*** value decreases. A reduced temperature difference reflects diminished temperature polarization and its associated impact on mass transfer in MD (e.g., ***α***~1, ***β***~1).

The effective ratio (***η***) for the mass transfer coefficient is defined as ***η*** = ***B*_b_**/***B*_s_**. This parameter reflects the impact of temperature polarization on mass transfer performance in membrane distillation, incorporating the effects of both ***α*** and ***β***. The following expression can be derived:

(7) $$J_{w}={\eta B}_{s}\left[\exp\left(23.1964-\frac{3816.44}{T_{f,b}-46.13}\right)-\exp\left(23.1964-\frac{3816.44}{T_{c,b}-46.13}\right)\right]$$

The intensity of temperature polarization is influenced by multiple factors, including bulk temperature, flow rate, and fluid disturbances, all of which directly affect the membrane surface temperature. Temperature polarization is an inevitable phenomenon during the operation of membrane distillation, leading to a discrepancy between the membrane surface temperature and the bulk temperature. A greater degree of temperature polarization results in a larger difference between ***B*_s_** and ***B*_b_**. The parameter ***η*** integrates various factors affecting the membrane surface temperature—such as bulk temperature, flow rate, and disturbances—and intuitively translates them into the impact of temperature polarization on mass transfer efficiency. Similarly to ***α*** and ***β***, the value of ***η*** reflects the intensity to which temperature polarization influences the overall mass transfer performance of the system. ***η*** ranges from 0 to 1, with values closer to 1 indicating a lesser impact of temperature polarization.

Theoretically, temperature polarization reduces this pressure difference as the driving force. At high temperatures, temperature polarization significantly affects the vapor pressure, while at low temperatures, the impact is negligible. The cold-side temperature polarization has an insignificant effect on the overall vapor pressure difference. Thus, the vapor pressure on the hot side can be approximated as the effective vapor pressure difference across the membrane [33].

This study focuses on characterizing the hot-side temperature polarization (***α***) and analyzing its impact on the MD process, providing insights for optimizing system performance.

## 4. Results and Discussion

### 4.1. Temperature Polarization near Membrane Surface

**Figure 2:**
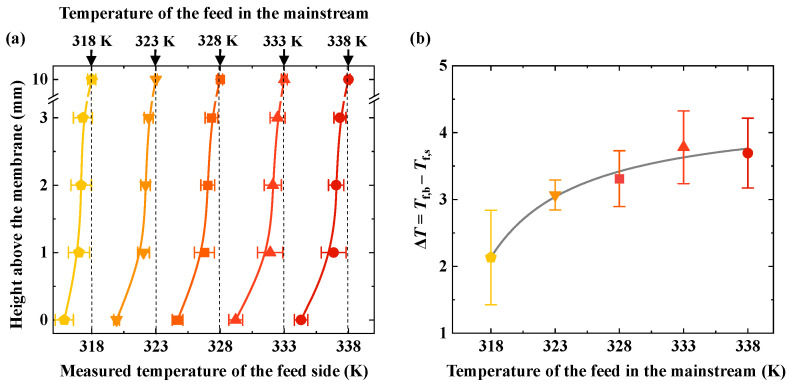
The impact of feed temperature on temperature polarization. (**a**) Measured temperatures at different heights above the membrane surface under varying bulk feed temperatures. (**b**) The temperature difference between the bulk feed and the membrane surface with increasing bulk feed temperature. ***T*_f,b_** − ***T*_f,s_** represents the temperature difference between the bulk feed and the membrane surface. The flow rate of the feed is set as 0.4 L·min^−1^. The temperature of the coolant is set as 299 ± 2 K. The coolant flow rate is set as 0.4 L·min^−1^.

The experimental results show that the feed temperature gradually decreases as the distance from the membrane surface decreases under various temperatures of the bulk feed (Figure 2a). The temperature of the feed decreases as it approaches the membrane surface. For example, when the feed temperature in the bulk flow region is 338 K, the temperature decreases by approximately 0.3 K as the distance from the membrane surface decreases from 3 mm to 2 mm. From 2 mm to 1 mm, the temperature decreases by about 0.2 K, and from 1 mm to 0 mm, the temperature drops by approximately 2.6 K. As shown in Figure 2b, the temperature difference increases with rising bulk feed temperatures across various feed conditions.

The test results indicate the presence of temperature polarization in the MD process. Temperature polarization occurs in the near-membrane interface region and is a typical near-membrane interface phenomenon. In this study, temperature polarization primarily exists in the region with a height of 0–3 mm from the membrane. The higher the feed temperature in the bulk flow region, the greater the temperature difference, indicating that temperature polarization becomes more pronounced as the feed temperature increases. This is because, as the feed temperature increases, the transmembrane flux increases exponentially. When the volatile component (typically water) in the feed evaporates at the membrane surface, it absorbs more heat, leading to an increase in the temperature difference. Hijaz et al. pointed out that in locations where the feed flow is relatively stable, the closer it is to the membrane surface, the greater the difference between the feed temperature and the bulk temperature [13]. Moreover, as the feed temperature increases, temperature polarization becomes more pronounced. We observed the same phenomenon in our study. Additionally, we found that in AGMD, the temperature change mainly occurs within a range of 0–1 mm from the membrane surface, where a larger temperature gradient exists.

Figure 2b shows the temperature difference between the membrane surface (***T*_f,s_**) and the bulk (***T*_f,b_**) at different bulk feed temperatures. As the bulk temperature increases, the temperature difference between the membrane surface and the bulk continues to increase. Furthermore, as the bulk temperature rises, the temperature difference increases rapidly at first; then the rate of increase gradually levels off. At a bulk feed temperature of 318 K, the temperature difference between the membrane surface and the bulk is 2.1 K. At a bulk feed temperature of 333 K, the temperature difference reaches its maximum, with a value of 3.8 K. When the bulk feed temperature is further increased to 338 K, the temperature difference no longer continues to increase. As the bulk temperature rises, the temperature difference increases, and temperature polarization becomes more significant. However, when the bulk temperature increases to a certain intensity, the temperature difference no longer increases. This may be due to the AGMD configuration used in this study, where there is an air gap between the membrane and the coolant. The membrane surface temperature increases with the bulk temperature, while the temperature difference tends to level off, as shown by the bulk feed and membrane surface temperatures in Figure 2a.

### 4.2. Temperature Polarization Factors

Common MD modules are made of plastic with low thermal conductivity, allowing heat exchange with the environment to be neglected during the process. The bulk temperatures are typically selected within a certain range, with cold-side and hot-side temperatures varying between 293 K and 303 K and 313 K and 363 K, respectively. In this study, the bulk temperatures on the cold and hot sides ranged from 297 K to 301 K and 318 K to 338 K, respectively, which falls within the commonly used range. Temperature polarization occurs adjacent to the membrane on both the feed and permeate sides, as evidenced by the experimental results showing a clear difference between the membrane surface and bulk fluid temperatures. The extent of this effect varies with operating conditions.

**Figure 3:**
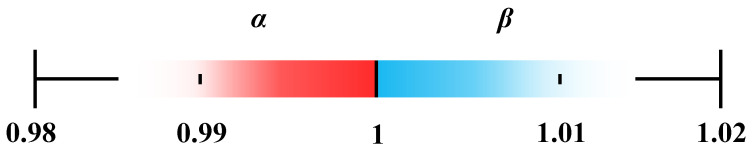
The typical ranges of temperature polarization factors, ***α*** and ***β***. In this study, the bulk feed temperature (***T*_f,b_**) is set 318 K to 338 K.

A proof-of-principle analysis is used to demonstrate the relationships between temperature polarization factors and temperature boundary ranges. ***α*** is the hot-side temperature polarization factor. When the hot-side bulk temperature equals the membrane surface temperature, no temperature polarization occurs on the hot side, yielding a maximum ***α*** value of 1. When the temperature difference across the membrane is at its maximum (***T*_f,b_** = 363 K; ***T*_c,b_** = 293 K), temperature polarization on the hot side becomes the most significant. Under such conditions, it can be assumed that the feed temperature at the membrane surface is close to the cold-side temperature (***T*_f,s_** = ***T*_c,b_** = 293 K), resulting in a minimum ***α*** value of 0.8. Based on this analysis, the range of ***α*** is from 0.8 to 1. Similarly, the range of the cold-side temperature polarization factor (***β***) is from 1 to 1.2. However, such a wide range of temperature variation was not observed in the actual experiments. According to the experimental data presented in the previous section, the minimum value of ***α*** occurred under a bulk feed temperature (***T*_f,b_**) of 338 K, where the corresponding membrane surface temperature (***T*_f,s_**) of the feed was 334.3 K. Under these conditions, ***α*** was 0.99, indicating that the variation in α under practical conditions is minimal. Since the influence of the cold side is very limited, the maximum value of ***β*** was only 1.01, as shown in Figure 3.

### 4.3. Effects of Feed-Side Temperature Polarization on Transmembrane Flux and Mass Transfer Coefficient

**Figure 4:**
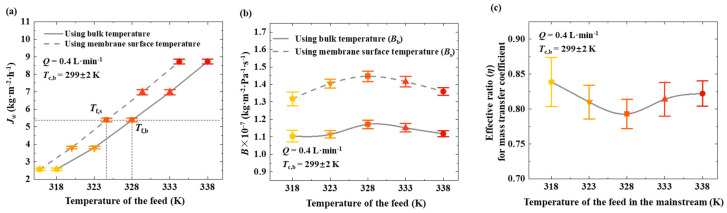
Analysis of mass transfer coefficient and effective ratio under varying bulk feed temperatures. (**a**) Comparison of mass transfer coefficients calculated using bulk feed temperature (***B*_b_**) and membrane surface temperature (***B*_s_**). (**b**) Temperature difference between bulk feed and membrane surface, corresponding to each condition. (**c**) Effective ratio (***η***) for mass transfer coefficient under different bulk feed temperatures. Results show that ***η*** remains within narrow range (approximately 0.75–0.85), indicating consistent impact of temperature polarization under tested conditions in this study. Coolant flow rate is set as 0.4 L·min^−1^. The varying colors of the markers represent distinct temperatures.

Figure 4a shows the transmembrane flux at different bulk feed temperatures. In this work, the temperature of the feed at the membrane surface was also measured. This study shows the variation in transmembrane flux with changes in bulk feed temperature and membrane surface temperature. The bulk temperature is always higher than that near the membrane surface, indicating that the presence of temperature polarization reduces transmembrane mass transfer performance. For instance, when the bulk feed temperature is 328 K, the transmembrane flux reaches 5.4 kg·m^−2^·h^−1^, while the temperature at the membrane surface is only 324.6 K. In the absence of temperature polarization, heating the feed to 324.6 K would be sufficient to achieve the same flux. This demonstrates that temperature polarization leads to the ineffective utilization of the supplied heat. Figure 4b presents the mass transfer coefficient calculated using the bulk feed temperature (***B*_b_**) and that calculated using the membrane surface temperature (***B*_s_**). The fluctuations in coefficients across different bulk feed temperatures are relatively small, allowing them to be considered nearly constant. The ***B*_b_** value (1.16 × 10^−7^ kg·m^−2^·Pa^−1^·s^−1^) is smaller than the ***B*_s_** value (1.43 × 10^−7^ kg·m^−2^·Pa^−1^·s^−1^), indicating that temperature polarization suppresses the mass transfer across the membrane. Figure 4c shows the effective ratio (***η***) for the mass transfer coefficient corresponding to different bulk feed temperatures. Under the conditions of this study, the impact of temperature polarization falls within a relatively narrow range, with ***η*** values approximately between 0.75 and 0.85.

### 4.4. Enhancing MD Performance by Suppressing Feed-Side Temperature Polarization

To minimize the impact of flow irregularities on temperature measurements, the feed flow rates were set to 0.4 L·min^−1^, 0.8 L·min^−1^, and 1.2 L·min^−1^, corresponding to Reynolds numbers (***Re***) of 170, 340, and 510, respectively. The coolant flow rates were also set to 0.4, 0.8, and 1.2 L·min⁻^1^. The Reynolds number is calculated as follows [31]:

(8) $$Re=\frac{\rho u_{av}d_{h}}{\mu }$$

where ***ρ*** is the fluid density, ***u*_av_** is the average velocity, ***d*_h_** is the hydraulic diameter, and ***μ*** is the viscosity of the fluid.

**Figure 5:**
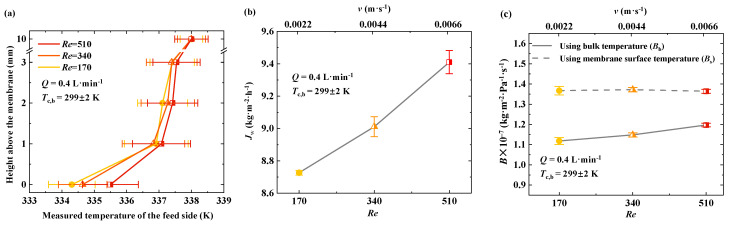
Influence of feed flow characteristics on temperature polarization near membrane surface on hot side. (**a**) Measured temperature differences between membrane surface and bulk feed at various flow velocities. Temperature variations from bulk region to near-membrane region under different Reynolds numbers at fixed bulk feed temperature of 338 K. (**b**) Relationship between transmembrane flux values and Reynolds number (***Re***). (**c**) Variation in ***B*_s_** and ***B*_b_** with Reynolds number. ***B*_s_** is calculated based on membrane surface temperature on feed side, while ***B*_b_** is calculated using bulk feed temperature.

The feed temperature was maintained at 338 K, and the feed flow rate was increased from 0.4 L·min^−1^ (***Re*** = 170; ***v*** = 0.0022 m·s^−1^) to 1.2 L·min^−1^ (***Re*** = 510; ***v*** = 0.0066 m·s^−1^). The temperature distribution of the feed at different heights is shown in Figure 5a. In the region 0–1 mm from the membrane surface, the feed temperature shows a noticeable increase with an increase in flow rate. At a flow rate of 0.4 L·min^−1^, the membrane surface temperature is 334.3 K, while at 1.2 L·min^−1^, it increases to 335.5 K. In the 1–3 mm region from the membrane, the feed temperature gradually decreases with increasing distance from the membrane surface. However, changes in the feed flow rate do not significantly affect the feed temperature in this region. At a height of 10 mm, the flow rate no longer influences the feed temperature. The effect of flow rate on the feed temperature is the most significant in the 0–1 mm region from the membrane surface. In the 1–3 mm region, the feed temperature remains within the range of 337–337.5 K, with only a minor increase.

For the AGMD configuration, the temperature difference between the membrane surface and the bulk increases rapidly at first, then more slowly, as the feed temperature rises. Once the feed temperature exceeds a certain value, the temperature difference between the feed and the membrane surface stabilizes. At this stage, the loss in the transmembrane mass transfer driving force due to temperature polarization does not continue to increase. The flow rate mainly affects the temperature of the feed in the 0–1 mm region from the membrane surface.

MD is a coupled process involving flow, heat transfer, and mass transfer. The change in the feed flow regime near the membrane interface affects both convective heat transfer and mass transfer, which in turn influences transmembrane mass transfer. Increasing the feed flow rate is a simple and effective method for suppressing temperature polarization. The flow regime within the module can be measured using the Reynolds number, which increases as the flow velocity increases. In this study, thermocouples were used to monitor the feed temperature at different heights above the membrane. To minimize the impact of flow irregularities on temperature measurements, we assume that the flow remains laminar throughout the experiments based on the Reynolds numbers. By increasing the feed flow rate while maintaining constant temperatures on both the hot and cold sides, the variations in transmembrane flux (***J*_w_**) and the mass transfer coefficient (***B***) with flow rate were obtained (Figure 5b,c). The results indicate that both ***J*_w_** and ***B*_s_** increase with increasing feed flow rate. At a flow rate of 0.4 L·min^−1^ (***Re*** = 170), ***J*_w_** and ***B*_s_** were 8.7 kg·m^−2^·h^−1^ and 1.12 × 10^−7^ kg·m^−2^·Pa^−1^·s^−1^, respectively. When the flow rate increased to 1.2 L·min^−1^ (***Re*** = 510), ***J*_w_** reached 9.4 kg·m^−2^·h^−1^, and ***B*_s_** increased to 1.21 × 10^−7^ kg·m^−2^·Pa^−1^·s^−1^.

According to fluid dynamic theory, as the feed flows over the membrane surface, a velocity boundary layer forms. During the MD process, both temperature and concentration boundary layers also develop on the membrane surface. The thickness of these boundary layers is related to the flow rate. Increasing the flow rate reduces the velocity boundary layer thickness, thereby enhancing heat and mass transfer, mitigating temperature polarization, and improving transmembrane mass transfer performance. Figure 5 illustrates that higher feed flow rates increase the transmembrane mass transfer coefficient, indicating the mitigation of temperature polarization and improved mass transfer performance in membrane distillation. The experimental results are consistent with those reported by Ali et al. [14], confirming that an increase in the Reynolds number enhances heat transfer efficiency from the bulk feed to the membrane surface. This improvement reduces the extent of temperature polarization, thereby leading to an increased transmembrane flux and mass transfer coefficient.

Based on the analysis above, both the hot-side temperature polarization factor (***α***) and the cold-side temperature polarization factor (***β***) and the effective ratio (***η***) for the mass transfer coefficient are influenced by various factors, including feed temperature, flow rate, height from the membrane surface, and channel height. In general, increasing the flow rate, reducing channel height, and increasing disturbances to the feed can enhance turbulence near the membrane interface, reduce temperature polarization, and increase transmembrane flux.

### 4.5. Effective Ratio for Mass Transfer Coefficient

**Figure 6:**
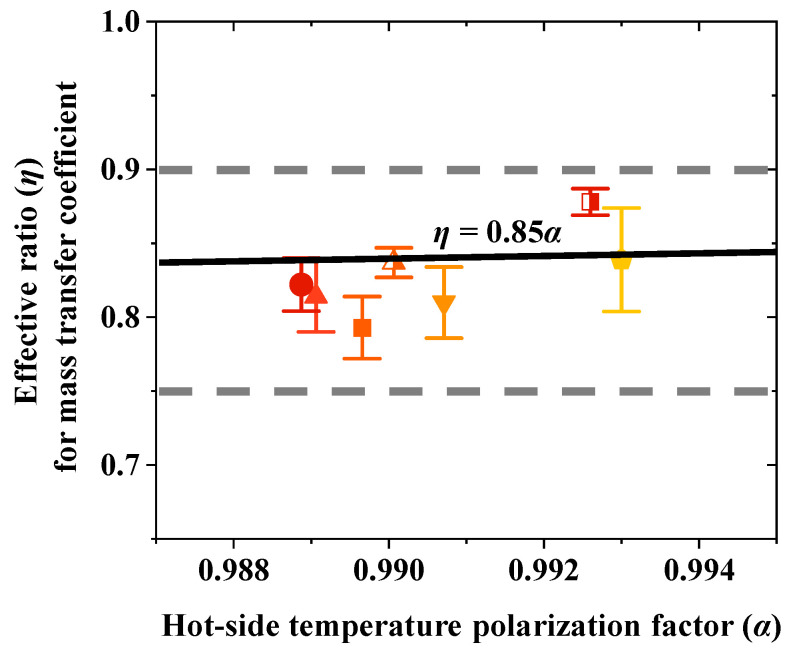
Relationship and distribution range of effective ratio (***η***) for mass transfer coefficient and hot-side temperature polarization factor (**α**) under typical MD operating conditions.

Based on the experimental data obtained in this study, multiple sets of values for ***α*** and ***η*** were calculated. Overall, the values of ***η*** consistently fell within a specific range, approximately between 0.75 and 0.90 (Figure 6). The effective ratio (***η***) for the mass transfer coefficient is primarily influenced by the hot-side temperature polarization factor (***α***). Under typical membrane distillation operating conditions, a linear relationship between ***α*** and ***η*** can be established as follows:

(9) η=0.85α

The factor ***α*** reflects all potential influences on the temperature polarization on the hot side. Increasing the flow rate can effectively reduce the degree of temperature polarization, thereby increasing both ***α*** and ***η***. As indicated in the previous section, increasing the flow rate from 0.4 L·min⁻^1^ to 1.2 L·min⁻^1^ improved **α** from 0.989 to 0.993 and ***η*** from 0.82 to 0.88. The varying colors of the markers represent distinct temperatures. The upper and lower doted lines in the figure indicate the general range of the data points.

## 5. Conclusions

This experimental investigation into temperature polarization in an air gap membrane distillation system provided critical insights into its impact on transmembrane mass transfer performance. By employing precise T-type micro-thermocouples, this study successfully monitored temperature gradients near the membrane surface, revealing that temperature polarization is the most pronounced within 0–1 mm from the membrane, with temperature differences increasing as bulk feed temperatures rise (up to 3.8 K at 333 K). The results confirm that temperature polarization reduces the effective temperature difference across the membrane, leading to a significant suppression of transmembrane flux and mass transfer efficiency, as evidenced by the discrepancy between the mass transfer coefficients calculated using bulk and membrane surface temperatures. The introduction of temperature polarization factors provided a robust framework for quantifying the impact of temperature polarization. *α* ranges from 0.99 to 1, while ***β*** ranges from 1 to 1.01. Increasing the flow rate enhances both the transmembrane flux and the mass transfer coefficient. The effective ratio for the mass transfer coefficient is introduced to quantify the extent to which mass transfer is affected by temperature polarization. In this work, ***η*** values were generally distributed in the range of 0.75 to 0.90. The value of ***η*** is primarily influenced by the hot-side temperature polarization factor, and a relationship of ***η*** = 0.85***α*** is proposed. By precisely measuring temperatures near the membrane surface and introducing metrics, this research clarifies how temperature polarization reduces transmembrane flux and mass transfer efficiency, bridging the gap between theoretical and experimental performance. Based on the approximate range of the temperature polarization layer in AGMD, system design can focus on reducing temperature polarization in this region to improve performance. This enhances membrane distillation modeling and optimization, critical for applications like desalination and wastewater treatment. It provides a straightforward engineering approach to estimating system performance. This is particularly important for large-scale MD systems, which involve numerous complex influencing parameters. The proposed empirical relationship and evaluation parameters (***α***, ***β***, and ***η***) offer a simple and practical tool for quickly assessing system performance and identifying optimization potential in large-scale applications.

## Data Availability

The data supporting the conclusions of this article will be made available by the authors on request.
